# Navigating mental health issues: exploring cultural causal attribution, stigmatization, and care recommendation behavior in Pakistani adolescents and young adults

**DOI:** 10.1186/s40359-025-03337-0

**Published:** 2025-08-27

**Authors:** Rizwan Abbas, Piet Bracke, Katrijn Delaruelle

**Affiliations:** 1 Health and Demographic Research (HeDeRa), Department of Sociology, Ghent University, Ghent, Belgium; 2 Department of Sociology, Ghazi University Dera Ghazi Khan, Dera Ghazi Khan, Punjab Pakistan

**Keywords:** Mental health issues, Causal attribution, Help-seeking care recommendation, Perceived public stereotypical attitude, Adolescent and young adults

## Abstract

**Background:**

Evidence has shown that the use of mental health services is less common among Pakistani adolescents and young adults compared to their peers in Western and English-speaking countries, despite a higher prevalence of mental health issues. This disparity suggests the presence of cultural and societal stigma affecting help-seeking behaviors. Our study aims to explore the prevalent forms of mental health causal attributions and help-seeking care recommendations in this population. Additionally, we investigated how these causal attributions influence help-seeking care recommendations and in what way perceived stigmatizing attitudes play a mediating role.

**Methods:**

The self-administered cross-sectional study included 1,328 college undergraduates aged 15–24 years in Layyah, Pakistan. Causal attributions were measured using 28 items distributed into five groups. Help-seeking care recommendations were assessed on a 20-item scale divided into four groups. Public stigmatizing attitudes were measured using items from the Eurobarometer 64.4 survey. The effect of causal attributions on help-seeking care recommendations was analyzed through multiple linear regression. Finally, we estimated the mediation effect of perceived public stigmatizing attitudes using PROCESS v4.0 macros for SPSS.

**Results:**

More than half (51.7%) of the adolescents demonstrated stereotypical attitudes, and one in four underestimated the severeness of the complaints. Psychosocial causes were the most prevalent mental health attribution, identified in 98% of the responses. Similarly, self-care (97.8%) and informal social support (97.7%) were the most common help-seeking care recommendations. Our findings revealed that labeling mental health issues as having religious and supernatural causes relates to formal social support from religious healers [β = 0.23, 95% CI 0.17–0.29, *p* < 0.001], and participants who attributed psychosocial causes also recommended support from religious healers [β = 0.19, 95% CI 0.10–0.27, *p* < 0.001]. The mediation model indicated an indirect association consistent with a partial mediating role of perceived public stereotypical attitudes, as adolescents and young adults attributing psychosocial causes to mental health were more likely to recommend formal social support from religious healers (β = 0.31, *p* ≤ 0.001) and informal social support (β = 0.29, *p* ≤ 0.001).

**Conclusion:**

The study confirmed that attributing causes to mental health strongly relate to care recommendation patterns. Furthermore, the mediating role of perceived public stereotypical attitudes was recognized in determining the relationship between mental health causal attribution and help-seeking care recommendations.

**Supplementary Information:**

The online version contains supplementary material available at 10.1186/s40359-025-03337-0.

## Background

Adolescence constitutes a pivotal stage of development [1, 2], during which biological changes and societal influences shape the mental well-being of individuals [3]. The mental health issues faced by adolescents have profound and far-reaching implications for both individuals and society at large. These issues, stemming from emotional turmoil, the navigation of developmental milestones, and the pressure of societal factors such as economic impact, identity formation, and peer pressure, create a complex web of vulnerabilities that predispose adolescents to mental health issues [4, 5].

The pervasive issue of mental health among adolescents and young adults is a significant public health concern. Research indicates that approximately one in five adults experience psychiatric vulnerabilities before the age of 25 [6]. Moreover, epidemiological studies from Western and English-speaking countries reveal that 10–15% of adolescents do not seek help for mental disorders [7, 8]. However, there is a lack of empirical data regarding help-seeking behavior for mental health issues among Pakistani adolescents and young adults, despite the higher prevalence of mental health issues within this population. For instance, research indicates that one-third of the adolescents in Pakistan experience socio-emotional issues [9]. A study identified a 31.4% prevalence of suicidal ideation among young Pakistani adolescents and young adults [10]. Furthermore, studies have found a 29% prevalence of mental disorders among young Pakistanis [11], with high rates of depression and anxiety [12, 13], as well as academic stress [14, 15]. Despite the higher prevalence of mental health issues among adolescents in Pakistan, the utilization of mental health services remains significantly lower compared to the global average [16, 17].

Attributing causes to mental health issues have significant impact on individuals’ help-seeking behavior [18–20]. Causal attributions refer to the beliefs individuals hold regarding the origins of mental health issues, which can range from biological and psychological factors to environmental and supernatural causes [16, 21]. These beliefs significantly influence the type of care individuals are likely to seek. For example, a recent study on causal attributions and help-seeking care recommendations towards mental health indicates that personality attribution is the most impairing belief, leading to lower openness towards psychological issues and more stigma, while psychosocial attribution is associated with higher help-seeking [19]. Other studies revealed that attributing mental health issues to biomedical causes increases the perceived need for professional help and help-seeking intentions, especially among individuals with no prior treatment experiences [21, 22].

Perceived stigma may act as a mediator in the relationship between causal attributions and help-seeking care recommendations [23]. This phenomenon can be partially attributed to the unfavorable attitudes of Pakistani adolescents and young adults towards seeking professional psychiatric treatment, as they often turn to religious and spiritual healers that act as formal sources for help and informal sources of help such as family and friends [24, 25]. This mediating role of stigma highlights the complex interplay between causal attribution and help-seeking care recommendations, underscoring the need for stigma reduction efforts to improve mental health service utilization. However, there is a dearth of empirical research within Pakistan that explains the relationship between public stigma and help-seeking attitudes among adolescents and young adults. While several studies worldwide, especially in Western countries, have investigated the relational aspects of help-seeking behavior and public perceived stigma, this aspect is subject to limited research in Pakistan.

Given this context, this study makes several significant contributions to the existing body of literature. It is the first attempt to consider the implications of causal attribution for care recommendation behavior within the context of Pakistani adolescents and young adults. While recent empirical studies in Pakistan have focused on the exploration of supernatural healing practices and traditional interpretations of illness [26, 27], the present study takes a broader approach by not only considering traditional cultural aspects but also focusing on the bio-psychosocial characteristics that contribute to a more comprehensive understanding of mental health issues. By combining modern approaches with cultural practices, this study offers a better framework for studying mental health issues, rationalizes the approach of healthcare practitioners, and enhances early-stage identification of mental health issues.

## Theoretical and conceptual framework

### Causal attributions and Help-seeking care recommendation

There are different approaches that explain the causal attribution of mental health issues and its relation to help-seeking care recommendations. In this study, we need to consider the perceptions of adolescents and young adults through their cultural, social, and structural dynamics. To comprehensively study this phenomenon, we turn to the illness model that includes biomedical, psychological, socio-cultural, and biopsychosocial models. Additionally, causal attribution theory offers a framework to understand the causal explanation of mental health issues, its influence on help-seeking care recommendations, and the mediating role of perceived stigma [19, 21, 28, 29].

First, the biomedical model attribution mental health issues to genetic inheritance, brain injuries, and neurotransmitter imbalances [30, 31]. The primary emphasis of the biological model is on medical interventions, recommending the consultative role of psychiatrists and medical treatments as help-seeking care recommendations [32–34]. Second, the psychological model explains trauma and dysfunctional behavior as the leading causes of mental health issues [35]. This model emphasizes various forms of psychotherapy, including cognitive behavioral therapy (CBT) at the micro level, with psychiatrists and counselors as help-seeking recommendations [35]. Third, the sociocultural model [36] discusses socioeconomic factors, societal pressure, and cultural stigma as causal attribution of mental health issues [37]. Proponents suggest macro-level approaches such as community support programs, counseling adherent to societal and cultural norms, and addressing systemic issues at the institutional level as care recommendations [38, 39]. Fourth, the biopsychosocial model [40] provides a holistic approach by combining biological, psychological, and sociocultural factors to understand mental health issues. According to this model, mental health issues result from genetic predispositions combined with cognitive and emotional dysfunction and socio-environmental influences [41, 42]. It recommends a comprehensive approach to care, where proponents recommend medication, psychotherapy, and social support systems as help-seeking care recommendations [34, 43].

Lastly, causal attribution theory [44, 45] infers that an individual’s causal explanation of illness depends on the available information and cues in society, which subsequently influence their attitudes and behaviors towards those experiencing illness [46, 47]. According to the proponents of causal attribution theory, internal and external attributions are two forms of attributions that lead individuals to influence their attitudes towards mental health issues [48]. Internal attributions are the outcome of personality traits and individual choices, while external attributions result from socioeconomic conditions and life events [49, 50]. For instance, attributing external factors to mental health issues stemming from surroundings increases the likelihood of recommending healthcare interventions. On the other hand, when individuals explain mental health issues as stemming from internal factors such as personal weakness and moral failings, they are more likely to recommend seeking help from informal networks and self-help strategies [51, 52]. The presented models show that attributing causes to mental health significantly influences help-seeking behaviors and can be exacerbate stigma.

### Stigma as mediator

Perceived public stigma acts as a mediator in the relationship between mental health causal attribution and help-seeking care recommendations, resulting from how causes are perceived and how individuals prefer to address mental health issues. For instance, in societies where mental health is highly stigmatized, individuals are reluctant to seek professional help and rely on informal social support and non-medical interventions [12, 16, 53]. Public stigma refers to the negative reactions, including stereotypical attitudes, prejudice, and discrimination, exhibited by the general population towards mental health [54]. Mental health stigma remains a significant global challenge [55] and has a wide range of negative consequences, including reduced help-seeking behavior among individuals facing mental health issues [56, 57]. This reluctance for seeking help is often driven by discrimination [58], fear of judgment [59], and social exclusion [60] associated with mental health issues [61, 62]. Consequently, individuals may turn to family, friends, or community leaders for support [63, 64], or they might seek alternative treatments such as spiritual healing [65, 66]. These preferences are influenced by cultural beliefs and the perceived acceptability of various treatment options within the community [67, 68]. The stigma surrounding mental health can thus significantly hinder the utilization of mental health services, leading to underreporting [69, 70] and untreated conditions [71], which further perpetuate negative outcomes [72, 73] for adolescents and young adults.

The specific aims of the study are to (a) explore the prevalent forms of mental health causal attribution and help-seeking care recommendations in adolescents and young adults; (b) examine how attributing causes to mental health issues relate to help-seeking care recommendation patterns; and (c) test the mediating role of perceived public stigma attribution.

## Methods

### Sampling and data collection

This cross-sectional study was conducted from November 2022 to February 2023 to investigate attitudes toward mental health and help-seeking behavior among Pakistani college students. The survey employed a self-administered approach and focused on public sector colleges within the Layyah district, situated in the southern region of Punjab, Pakistan. Layyah is divided into three administrative units known as tehsils: Layyah, Karor, and Chaubara. The Pakistani education system encompasses various levels, including primary schools (1st to 5th grade), middle schools (6th to 8th grade), high schools (9th to 10th grade), colleges (11th to 14th grade), and universities (13th grade onward). Additionally, the education structure is divided into public and private institutions.

The sample for this study was drawn using multistage cluster sampling. Initially, we selected 12 colleges (4 from each tehsil). Subsequently, we invited 1,800 students to participate in our survey, comprising 1,300 (72%) higher secondary level students representing the age range of 15 to 19 years and 500 (28%) bachelor (undergraduate) level students aged between 19 and 24 years. A total of 1,450 (80.5%) students participated in the survey. However, 122 students did not complete the survey and were excluded from the data analysis. Therefore, a total of 1,328 students completed the survey and were included in the data analysis. We conducted the survey in a classroom setting: students completed them privately with no teachers or college staff present, and no identifying information was collected. Students were informed, both verbally and in writing, that their responses would remain anonymous and confidential and would be reported only in aggregate. A pilot study with 100 adolescents and young adults likewise confirmed that the items were clearly understood and the questionnaire was workable; no respondents suggested changes to its content. These steps collectively helped to improve response accuracy despite the sensitive nature of the topic. Due to Covid-19 restrictions, classrooms were limited to 20 to 40 students per session. It was the first experience for the respondents to be part of such a survey.

Written vignettes related to depression were used to provide a common frame of reference for the students. These two vignettes (see Appendix A), depicting a female and a male character expressing mental health issues, were randomly assigned to the students.

The questionnaire used in the survey was adapted from the FWO Red Nose: Stigma Project [74] and was translated into Urdu using Euro-Reves forward-backward translation protocols [75]. The questionnaire was presented to the college students in both English and Urdu languages.

The Faculty of Political and Social Sciences at Ghent University, Belgium, provided ethical advice for the study vide reference number 2021/26/FSA. We obtained a permission letter from the Office of the Assistant Director of Colleges in Layyah to conduct the survey in the district’s colleges. Written consent was obtained from the students willing to participate in the survey. Participation in the survey was completely voluntary and did not involve any monetary benefits to the participants.

### Variables

The questionnaire used in this study was based on different validated scales and aimed to measure the approaches and attitudes of adolescents towards mental health issues. Causal-attribution items were adapted from the mental-illness causal-beliefs inventory of Martin et al. [76]. help-seeking recommendation items were taken verbatim from the Red Nose: Stigma Project inventory, which is derived from the General Help-Seeking Questionnaire and was recently re-validated in adolescents by McLaren et al. [77]. Lastly, perceived public stereotypes were assessed using the scale developed by Abbas et al. [78]. Both instruments have demonstrated strong internal consistency and test–retest reliability in previous studies.

#### Dependent variables (DV)

In our study, we assessed help-seeking care recommendations using a 20-item inventory organized into four categories: Informal Social Support (Involving parents, family member, friend, peers with similar experiences, a confidential teacher or class teacher, and fellow student), Formal Social Support from Religious Healers (religious healer, faith healer, traditional healer, and shamans), Formal Support from Health Professionals (medication, psychologist or psychiatrist, homeopathy or acupuncture, Doctor (general practitioner)), and Self-care (without doing anything, more self-discipline, strict upbringing, Praying, leisure activities, adjusting eating/sleeping habits). Recognizing the cultural specifics of Pakistan, we expanded the original inventory used in the FWO Red Nose: Stigma project by adding three items relevant to the local context. These additional items included consulting with a faith healer (amil/sinyasi), seeking remedies from a traditional healer (utilizing herbal, Unani/Greek medicine, or Hakeem), and obtaining treatment from shamans. Responses were gathered using a 4-point Likert scale ranging from “0 = No, Probably Not” to “3 = Yes, Definitely.” The overall reliability for the help-seeking care recommendations was α = 0.77 for the current sample. The subscale reliability was: 0.69 for informal social support, 0.57 for formal social support from religious healers, 0.52 for formal support from health professionals, and 0.41 for self-care. The comparatively low α for subscales reflects the deliberately broad set of self-management strategies included; items were chosen to tap diverse, loosely related behaviors rather than a single latent construct, so internal consistency was expected to be modest [79].

#### Independent variables (IV)

We assessed causal attributions of mental health using a 28-item inventory. This included 10 items focusing on beliefs commonly held in Pakistani society, divided into six categories: Socio-Economic Causes (less educated parents, father’s low wages, poverty, community disengagement, negative attitudes tied to the father’s occupation, family’s economic position), Biomedical Causes (mental disorders, brain disorders, genetic issues, drug use), Academic Stress (studies-related stress, fear of failure, unmet family expectations in academics, loss of community respect due to educational underachievement), Psychosocial Causes(loneliness, depression, major life events such as the death of a loved one, upbringing, conflicts with loved ones, exposure to violence, being bullied), Religious and Supernatural Causes (guilt, deviation from obligatory religious practices, perceived as divine retribution, beliefs in sorcery, ghosts, and witchcraft), Person-Centred Causes (developmental milestones in maturity, sleeping, eating habits, and sports habits). Each potential cause was rated on a 4-point Likert scale ranging from “0 = No, Probably Not” to “3 = Yes, Definitely” to gauge the student’s perception of each item. Regarding the reliability of the items in causal attribution, the overall alpha was α = 0.77 for the current sample, with the following subscale reliabilities: socio-economic causes at 0.64, biomedical causes at 0.50, academic stress at 0.60, psychosocial causes at 0.48, religious and supernatural causes at 0.52, and person-centred causes at 0.25. The alpha scores indicate the variation in response patterns, as the scale aims to encompass a wide range of mental health causal attribution rather than focusing on a unified construct. Thus, the subscale’s low α within the broader causal-attribution category is due to its intentionally heterogeneous items – such as sleeping habits, sports engagement, and developmental milestones – which do not form a unitary construct but collectively describe individual-level factors.

#### Mediating variable(s)

Perceived public stereotypical attitude was assessed using a 5-item scale (see study by Abbas et al. [78] for the list of items). The scale helped to identify the public perceived stigmatic attitude among college students. The responses of the students were taken on a 5-point Likert scale ranging from “No, definitely not” to “Yes definitely” (Cronbach’s α = 0.74). Higher stigma was identified by the higher score.

#### Control variables

Socio-demographic characteristics comprised age in years, gender (0 = male [ref. cat.], 1 = Female), Residential Area (0 = Rural [ref. cat.], 1 = Semi Urban, 2 = Urban), Family System (0 = Nuclear [ref. cat.], 1 = Joint, Extended), Father’s Profession (0 = Farmer [ref. cat.], 1 = Self-employed, 2 = Government Job, 3 = Private job, 4 = Daily wage worker).

### Statistical analysis

The description of the variables is provided in Table 1. Noting that the data exhibited signs of clustering, we initially assessed the association of care recommendations with causal attribution and the other socio-demographic variables using multiple linear regression analysis. The results, including unstandardized beta (β) coefficients, *p-*values, and the 95% confidence intervals (CIs), are presented in Table 2. Additionally, the study examined both the direct and indirect effects of causal attribution on care recommendations. The indirect effect mediated by public perceived stigma, were estimated using PROCESS v4.0 [80] macro for SPSS. The mediation model and its results are presented in Table 3 in the analysis section. All analyses were performed using SPSS version 26.0.

## Results

**Table 1:** Description of adolescent characteristics, and mediating variable (*N* = 1328)

	*N*	%
**Gender**		
Male	732	55.1
Female	596	44.9
**Residential Area**		
Rural	655	49.3
Semi Urban	330	24.8
Urban	343	25.8
**Family System**		
Nuclear	834	62.8
Joint	324	24.4
Extended	170	12.8
**Father’s Profession**		
Self-employed	295	22.2
Farmer	508	38.3
Government Job	224	16.9
Private job	109	8.2
Daily wage worker	192	14.5
	**Mean (SD)**	**Min. – max.**
Age	17.9 (1.7)	15–23
Severity of mental health condition	6.3 (3.2)	0–10
**Mediating Variable**		
Public perceived stigmatizing attitude	3.1 (0.9)	1–5

In the present study, the majority of the participants were male (55.1%), lived in rural areas (49.3%), hailing from nuclear family (62.8%), and with fathers as farmers (38.3%). The average age of the participants was 17.9 (1.7) years (Table 1).

**Table 2:** Participants percentage who agreed (yes probably/yes definitely) with each causal attribution and care recommendation for mental health issues (*n* = 1328)

Causal Attribution	*N*	%
**1. Socio-Economic causes**		
At least any of socio-economic causes	1261	95.0
All socio-economic causes	194	14.6
**2. Biomedical Causes**		
At least any of the bio-genetic causes	1118	84.2
All bio-genetic causes	120	9.0
**3. Academic Stress**		
At least any of the academic stress	1224	92.2
All academic stress	431	32.5
**4. Psychosocial Causes**		
At least any of the psychosocial causes	1301	98.0
All psychological causes	85	6.4
**5. Religious and supernatural causes**		
At least any of the religious and supernatural causes	1221	91.9
All religious and supernatural causes	126	9.5
**6. Person-centered causes**		
At least any of the person-centred cause	1083	81.6
All person-centred cause	548	41.3
**2. Care Recommendations**		
**1. Informal Social Support**		
At least one of the informal social support	1297	97.7
All informal social supports	489	36.8
**2. Formal Social Support from religious healers**		
At least one of the formal social support from religious healers	1038	78.2
All formal social support from religious healers	125	9.4
**3. Formal Support from health professionals**		
At least one of the formal social support from health professionals	1177	88.6
All formal social support from health professionals	199	15.0
**4. Self-Care**		
At least one of the self-care	1299	97.8
All sources of self-care	72	5.4

In our study, psychosocial factors emerged as the predominant causal attribution for mental health issues in adolescents and young adults, with 98.0% of participants agreeing that at least one of these factors played a significant role in the development of mental health issues (Table 2). Within the category of psychosocial issues, depression (75.7%) and feelings of loneliness (71.8%) were most cited as specific causes (see Appendix-B). The finding underscores the significance of psychosocial factors in shaping perceptions of mental health concerns among Pakistani adolescents and young adults (Table 2).

Furthermore, our study unveils distinct patterns in terms of care recommendations. Our sample demonstrated a higher propensity to self-care (97.8%) and informal social support (97.7%) as opposed to seeking formal support from healthcare professionals. Importantly, self-care practices such as prayers (81.7%) and adjustments to eating and sleeping habits (78.3%) were commonly reported mental health care recommendations. Furthermore, informal social support, including support from a confidential teacher or class teacher (76.7%), and fellow students (75.7%) featured prominently in the care recommendations put forth by Pakistani adolescents and young adults (see Appendix-B). This suggests a preference for personal coping strategies among adolescents and young adults and seeking support from within their social circles.

**Table 3:** The association between causal attribution and care recommendations behavior for mental health illness, controlled for socio-demographic characteristics (*N* = 1328)

Attribution	Perceived Public Stereotypes	Informal Social Support	Formal Social Support from Religious Healers	Formal support from health professionals	Self-Care
	β (95% CIs)				
Socio-economic causes	0.06 (-0.04 to 0.06)	0.1 (0.04 to 0.16)**	0.06 (-0.01 to 0.12)	0.06 (-0.004 to 0.13)	0.05 (0.01 to 0.1)*
Biomedical Causes	0.04 (-0.05 to 0.04)	-0.03 (-0.08 to 0.02)	0.07 (0.01 to 0.13)*	0.09 (0.03 to 0.14)**	0.02 (-0.02 to 0.07)
Academic Stress	0.04 (-0.05 to 0.04)	0.11 (0.06 to 0.16)***	-0.03 (-0.09 to 0.03)	0.02 (-0.04 to 0.07)	0.05 (0.004 to 0.09)*
Psychosocial Causes	0.19 (0.07 to 0.19)**	0.16 (0.08 to 0.23)***	0.18 (0.1 to 0.26)***	0.12 (0.04 to 0.2)**	0.06 (0.01 to 0.12)*
Religious and supernatural causes	0.11 (0.02 to 0.11)*	0.11 (0.05 to 0.16)***	0.22 (0.16 to 0.28)***	0.12 (0.06 to 0.18)***	0.16 (0.11 to 0.2)***
Person-centred causes	-0.03 (-0.1 to -0.03)	0.07 (0.03 to 0.11)***	0.05 (0.001 to 0.09)*	0.07 (0.03 to 0.12)***	0.1 (0.07 to 0.13)***
**Age**	-0.05 (-0.08 to -0.02)***	0.01 (-0.01 to 0.03)	-0.002 (-0.02 to 0.02)	-0.02 (-0.04 to 0.003)	0.002 (-0.01 to 0.02)
**Gender (** ***Ref. cat. Male*** **)**					
Female	0.23 (0.12 to 0.33)***	0.09 (0.02 to 0.15)**	-0.09 (-0.16 to -0.01)*	0.14 (0.07 to 0.21)***	-0.01 (-0.06 to 0.05)
**Residential Area (** ***Ref. cat. Rural*** **)**					
Semi Urban	-0.12 (-0.25 to 0.004)	0.15 (0.07 to 0.23)***	0.06 (-0.03 to 0.15)	-0.01 (-0.1 to 0.08)	0.06 (-0.01 to 0.12)
Urban	-0.18 (-0.32 to -0.05)**	0.07 (-0.02 to 0.15)	0.05 (-0.04 to 0.15)	-0.11 (-0.2 to -0.02)**	0.09 (0.03 to 0.16)**
**Family System (** ***Ref. cat. Nuclear*** **)**					
Joint	-0.15 (-0.27 to -0.03)*	0.04 (-0.03 to 0.12)	0.07 (-0.02 to 0.15)	0.06 (-0.02 to 0.14)	0.03 (-0.03 to 0.09)
Extended	-0.30 (-0.46 to -0.15)***	0.02 (-0.07 to 0.12)	0.17 (0.07 to 0.28)***	0.04 (-0.07 to 0.15)	-0.04 (-0.12 to 0.04)
**Father’s Profession (** ***Ref. cat. Farmer*** **)**					
Self-employed	-0.08 (-0.22 to 0.06)	-0.03 (-0.12 to 0.05)	-0.08 (-0.17 to 0.02)	-0.02 (-0.11 to 0.08)	-0.04 (-0.11 to 0.03)
Government Job	-0.08 (-0.24 to 0.07)	0.02 (-0.08 to 0.11)	-0.001 (-0.11 to 0.1)	0.01 (-0.09 to 0.12)	-0.01 (-0.09 to 0.07)
Private job	-0.14 (-0.34 to 0.06)	-0.02 (-0.14 to 0.11)	-0.04 (-0.17 to 0.1)	-0.07 (-0.2 to 0.07)	0.05 (-0.05 to 0.15)
Daily wage worker	-0.11 (-0.27 to 0.05)	-0.05 (-0.15 to 0.05)	-0.04 (-0.15 to 0.07)	-0.06 (-0.17 to 0.05)	-0.08 (-0.16 to -0.001)*
**Perceived Public Stereotypical Attitude**		0.02 (-0.02 to 0.05)	0.03 (-0.01 to 0.07)	-0.002 (-0.04 to 0.04)	0.05 (0.02 to 0.07)***

Not significant, P ≤ *0.05, **0.01. ***0.001

Multiple regression analysis revealed how specific causal attribution related to particular types of care recommendations for mental health issues while controlling for several demographic variables. Religious and supernatural causes were found to be associated with formal social support from religious healers [β = 0.23, 95% CI 0.17–0.29, *p* < 0.001]. Psychosocial causes also significantly related to formal social support [β = 0.19, 95% CI 0.10–0.27, *p* < 0.001]. Participants who attributed mental health to religious and supernatural causes and psychosocial factors were more likely to recommend seeking support from religious healers. Conversely, biomedical causes for mental health issues demonstrated a positive pattern. Participants who attributed mental health to biomedical factors were more inclined to recommend formal support from healthcare professionals, indicating that they perceived these issues as requiring medical interventions. Further, participants who were more likely to associate mental health issues with academic stress were more inclined to recommend informal social support.

Additionally, the influence of control variables on care recommendation help-seeking behavior was examined. Female respondents were more likely to recommend formal support from health professionals compared to their male counterparts. Respondents residing in semi-urban areas were more likely to rely on informal social support compared to Urban residents. Those from extended families were more likely to recommend formal social support from religious healers. The categories of participants’ fathers’ professions had a negative association with all listed care recommendation groups. Age and perceived severity also significantly related to the attribution of self-care.

**Fig. 1:**
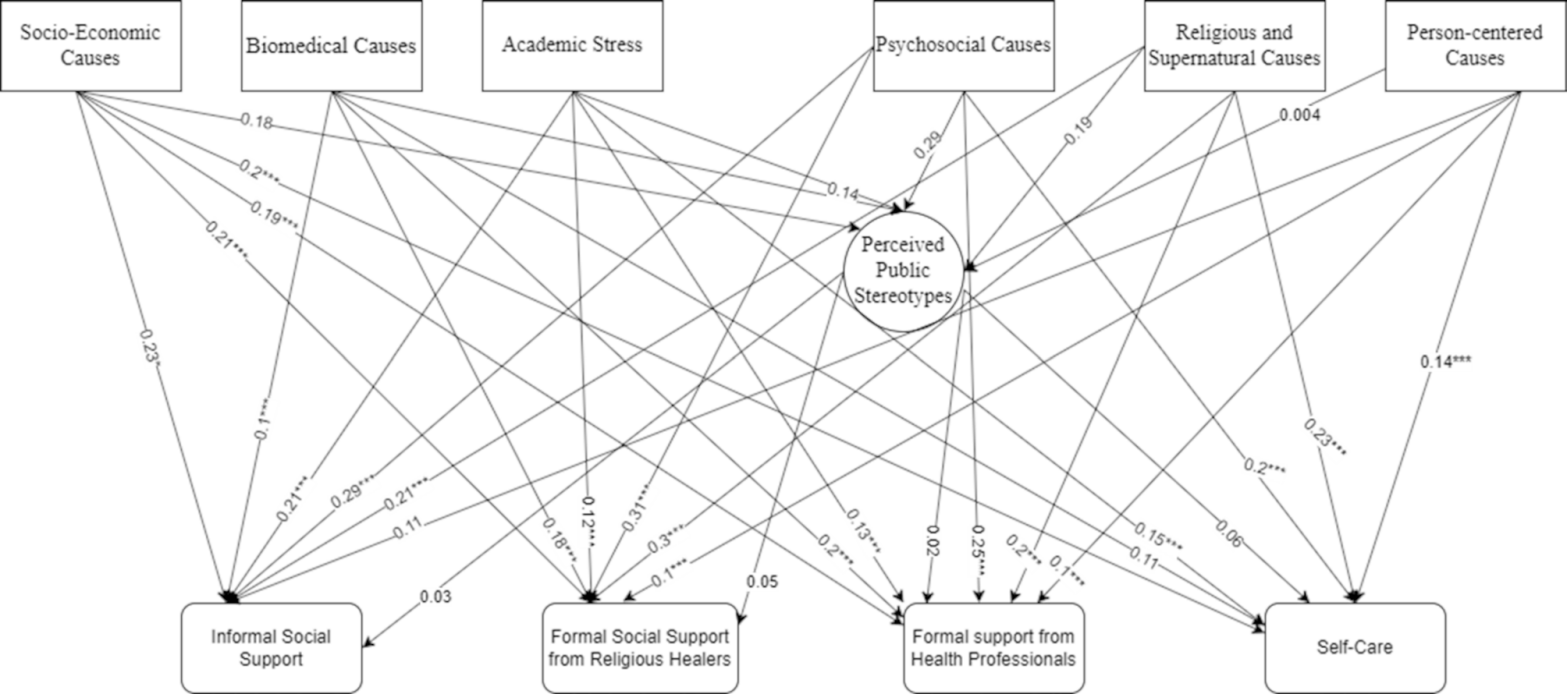
Direct and indirect relations between causal attribution and the care recommendation of mental health issues through perceived public stigmatic attitude Not significant, P ≤ *0.05, **0.01. ***0.001

Figure 1 presents the indirect relations between various causal attribution different types of help-seeking care recommendations, mediated by perceived public mental health stigma. This enables us to grasp a firm idea about the most prevalent form of mental health causal attribution relating to help-seeking care recommendations and the partial mediating role of perceived public stereotypes. The analysis identified that psychosocial factors, religious, and supernatural causes significantly relate to all types of help-seeking care recommendations.

The total relationship between psychosocial causes (independent variable) and all types of help-seeking care recommendations (dependent variable) confirms that attributing psychosocial causes to mental health significantly relates to recommendations for seeking help. More specifically, the coefficients suggest that adolescents and young adults attributing psychosocial causes to mental health issues are more likely to recommend formal social support from religious healers (β = 0.31, *p* ≤ 0.001) and informal social support (β = 0.29, *p* ≤ 0.001). Additionally, individuals who attributed mental health issues to religious and supernatural causes (independent variable) are more likely to recommend formal social care from religious healers (β = 0.29, *p* ≤ 0.001) and self-care (β = 0.29, *p* ≤ 0.0001).

The internalization of stigma was significantly associated with various forms of mental health support: informal social support (β = 0.03, *p* ≤ 0.02), formal social support from religious healers (β = 0.04, *p* ≤ 0.02), formal support from health professionals (β = 0.0103, *p* ≤ 0.02), and self-care (β = 0.1, *p* ≤ 0.02). Thus, the results of the mediating model demonstrate a significant indirect association (Fig. 1). Although the coefficients are not as large as those for psychosocial factors and religious causes, attributing causes to mental health significantly relate to the perceived public stigma, which in turn influences the type of help-seeking care recommendations (see Table 1 in supplementary material for a tabular presentation).

Additionally, among the sociodemographic characteristics, age and gender have varying significant effects across different support types. Females are generally more likely to suggest formal support from health professionals but less likely to advise seeking help from religious healers. Age shows a significant relationship with causal attribution for informal social support and self-care. The residential area indicates that respondents living in urban locations generally showed less influence of causal attribution on getting formal support from health professionals (see Table 1 in supplementary material).

## Discussion

This study revealed a significant association between causal beliefs and help-seeking care recommendations. Furthermore, the data indicated how internalization of public perceived stereotypes can play a mediating role between causal attribution and help-seeking care recommendations.

### Common causal attributions and Help-Seeking care recommendations

We found that half of the individuals surveyed held perceived stereotypical attitudes, and one fourth of the participants underestimated the severeness of the complaints. Psychosocial causes were the most endorsed causal attributions for mental health among adolescents and young adults in Pakistan. Similar findings have been observed in other studies conducted in Arab [81, 82] and Asian [83–88] countries. Pakistani adolescents were more likely to endorse psychosocial causation than Western populations [89]. The second most reported attributions were socio-economic causes. Stigma associated with lower socio-economic status and mental health stigma can lead to social exclusion, negatively impacting students’ mental health [90–93]. At the same time, academic stress was indicated by large number of respondents, which is unsurprising given that the participants were college students. This evidence supports other studies highlighting the impacts of academic-related stress on the mental health of adolescents [94–97]. Attributing mental health problems to academic stress might be due to lack of psychological education in the Pakistani curriculum until higher secondary levels, which may contribute to mismanagement of mental health issues and an increase in misconceptions and stigma among adolescents and young adults.

We also aimed to find out the prevalent forms of help seeking care recommendations. Participants primarily suggested self-care and informal social support for mental health issues, which is consistent with other studies [98, 99] and may reflect the limited availability of mental health services in Pakistan [11, 26]. Nevertheless, most of the participants recommended formal support from health professionals. This may be due to the consideration of college students for the study, and they indicated an increased awareness of the complexity of mental health issues. These findings are in line with the study of Li et al., [85] and Tawiah, Adongo & Aikins [100]. These findings reflect an improved understanding of mental health issues among adolescents and young adults. Finally, it was notable that about three in four participants believed that getting formal social support from religious healers could overcome the illness. Similar findings have been reported by other Pakistani studies that highlighted the critical role of traditional healers in mental health care pathways [24, 53]. Reliance on religious healers underscores the significant influence of cultural and religious beliefs on mental health care preferences and the potential barriers to accessing conventional psychiatric services. This phenomenon is also documented in Asian literature [18, 101–106]. One of the reasons for the critical role of traditional healers in curing mental health is that they often adopt a holistic approach to care [107], addressing not only the symptoms but also explaining illness through social, cultural, and spiritual angles that are acceptable by society at large [108–110].

### Association between causal attribution and help-seeking care recommendations

Participants who attributed mental health to religious and supernatural causes preferred getting formal social support from religious healers, which is consistent with prior findings [111–113]. This preference may reflect that Western-oriented mental health services are not yet widely accepted and trusted among Pakistani adolescents and young adults [16], possibly due to cultural beliefs and fear of social isolation associated with using services perceived as stigmatized. People may prefer traditional healers due to the perceived social acceptability and alignment of the treatment procedure with local norms and beliefs [114, 115]. Assuming religious healers to counter mental health stigma bridges the gap between conventional mental health services and religious healing practices.

### Mediating role of perceived public stigmatizing attitude

Additionally, we found that perceived public stereotypical attitude partially – but only modestly – mediates the association between causal attributions and care recommendations among adolescents and young adults. Attributing mental health to psychosocial causes was associated with slightly greater endorsement of informal support and recourse to religious healers; however, the indirect effects suggest that other mechanisms remain influential. This indicates that people’s beliefs about mental health issues and recommendations for care are influenced by prevailing societal stereotypes. Adolescents and young adults showed agreement with internalizing stereotypes, such as viewing mental health as dangerous or unpredictable, which can have serious consequences for access to care [17, 116]. Nevertheless, alternative mediators, such as self-stigma, family expectations, or beliefs about treatment effectiveness, may better explain help-seeking decisions and warrant investigation in future work.

### Study strengths and limitations

To the best of our knowledge, the current study is the first attempt to examine a sample of Pakistani adolescents and young adults. Unlike previous studies with smaller sample size of adolescents and young adults, our study realized a relatively large sample size. Other improvements were made over previous studies, including the use of standardized scale and a theoretical lens to assess culturally related causal attribution, their direct effect on care recommendations and mediating role of public perceived stigma.

However, our study findings should be interpreted considering certain limitations. First, the cross-sectional design does not establish a true cause and effect relationship. As the data is cross-sectional, the observed indirect paths represent statistical associations that do not establish temporal precedence; therefore, no causal inference can be drawn [117]. Second, we limit our sample to individuals aged 15–24 years students attending public colleges in Layyah District, Punjab. Consequently, the findings may not generalize to adolescents and young adults enrolled in private institutions, to those who have left school or are employed, or to youth in other regions of Pakistan. Third, it was the first time for our sample to tackle this kind of survey questionnaire, which may have moderate social desirability bias [81, 118].

Fourth, the α values indicate that the items within those subscales are heterogeneous, capturing a breadth of behavior rather than a tightly unified dimension. Consequently, associations involving these subscales should be interpreted with caution, as measurement error may attenuate observed relationships. Future research can refine these item sets and re-evaluate their dimensionality, for example through exploratory or confirmatory factor analysis or item-response theory, to optimize reliability while preserving the culturally relevant breadth of content [119].

Lastly, the minimal effect of perceived public stigma on help-seeking recommendations should be cautiously interpreted. This minimal effect resulted from adding control variables to our analysis. Nevertheless, it has been proven in research that adding control variables enhance the internal validity of the study by limiting confounding and extraneous variables [120]. In this case, the minimal effect of public perceived sigma reflects a good relationship between causal attribution and help-seeking recommendations [120] and leads to drawing important conclusions.

### Theoretical, research and clinical implications

The results of the current study have significant implications for the future mental wellbeing of adolescents and young adults. First, the research implications derived from our search point to a dearth of knowledge due to the non-use of valid assessment measures. This highlights the need for more rigorous research using valid and reliable assessment tools in the field of mental health stigma among Pakistani adolescents and young adults. Second, adolescents and young adults are often a neglected proportion of society when it comes to research on culturally sensitive topics. Our study focused on adolescents and young adults from socio-economically vulnerable backgrounds who may be discouraged from seeking mental health services due to stigma. This underscores the importance of conducting further research on a larger scale in the country, especially in the areas where people are seeking mental health services. Third, our study highlights the almost negligible application of theoretical perspectives in recent literature on mental health in Pakistan. This study integrated theoretical models to study the interplay of causal attribution, stigma, and help-seeking care recommendations in Pakistan. This provides an extended theoretical framework offering a more comprehensive approach for future research on mental health stigma in the country, helping to bridge the theoretical application gap.

Finally, the present study provides evidence to understand the mechanism of mental health stigma and its influence on help-seeking care attitudes. This information can inform clinical approaches aimed at promoting mental health of adolescents and young adults. Similar to the findings of Fekih-Romdhane et al., [81] in 16 Arab countries, our study also suggests that stigma, culturally specific causal attributions, and negative help-seeking care recommendation patterns were prevalent in our sample. For instance, our findings revealed that adolescents and young adults attributing mental health to religious and supernatural causes recommended social support from religious healers, indicating the rooted existence of clerics in Pakistani society. This implies that, while institutional arrangements to address public stigma is effective in Western countries [121], these arrangements might not be as effective among adolescents in Pakistani cultural settings. The absence of anti-stigma programs at the college and community level is allowing mental health stigma to persist in Pakistani societal settings.

To address these issues, it is essential to improve help-seeking care recommendations and provide early access to mental health care that align with the general public’s beliefs about mental health causal attribution in the Pakistani societal context. Excluding traditional healing practices from Pakistani culture is not a practical idea, and it is suggested that mental health professionals should be familiar with help-seekers’ cultural context, customs, and beliefs. Another approach could be to integrate traditional healing strategies into the mental healthcare system [114, 122].

In brief, associations were observed between causal attributions and preferred help-seeking behaviours, with perceived public stereotypical attitudes contributing to a small yet significant indirect association. Culturally endorsed options, particularly religious healers, also appeared to play a central role. Accordingly, comprehensive, context-sensitive strategies that address broader sociocultural dynamics may support improved mental-health service utilization among Pakistani adolescents and young adults.

## Conclusion

Our research revealed a strong association between mental health causal attributions and help-seeking care recommendations. Additionally, we identified a mediating effect of public perceived stigma that influences patterns of help-seeking care recommendations. In summary, this study contributes to the understanding of mental health issues by integrating potential mediators into a single model to examine the associations between mental-illness attributions and help-seeking care recommendations. Unlike prior research, which often focused separately on causal factors or help-seeking recommendations, this study provides empirical insights into the practical mental well-being of adolescents and young adults. It also highlights the significant role of perceived public stigma as a mediator and offers a context-specific perspective by drawing from a socio-economically vulnerable area, where no similar surveys have been conducted before. This approach underscores the importance of addressing stigma to improve mental health service utilization in diverse contexts. We also believe that this study serves as a “VOICE” calling for the attention of public mental health practitioners in Pakistan to tailor their interventions in accordance with the culturally prevalent believes.

## Supplementary Information

Below is the link to the electronic supplementary material.

Supplementary Material 1

## Data Availability

No datasets were generated or analysed during the current study.

## Abbreviations

DV: Dependent Variables
IV: Independent Variables
Ref. cat.: Reference Category
CI: Confidence Interval
v4.0: Version 4.0
%: Percentage
SD: Standard Deviation
Min.: Minimum
Max.: Maximum
