# Supplementary material for: Navigating mental health issues: exploring cultural causal attribution, stigmatization, and care recommendation behavior in Pakistani adolescents and young adults
Source: BMC Psychol. 2025 Aug 27;13:976. doi: 10.1186/s40359-025-03337-0 (PMC12392498; doi:10.1186/s40359-025-03337-0)
Supplement: Supplementary file 1 — Supplementary Material 1 [file 40359_2025_3337_MOESM1_ESM.docx]

**Appendix**

**Appendix A: *Example Vignette of Ali/Ayesha***

*The vignettes of student(s) indicating signs of mental health illness was as follows:* Ayesha/Ali is a 19-year-old girl/boy. She/He is living with her/his parents in a village. She/He has been sad for the last couple of days. She/He has abandoned her/his usual routine of going to college, attending classes, playing games with friends, and following scheduled activities. She/He stopped seeing her/his friends. She/He wakes up in the morning with a passive feeling and remains sad all day.

She/He has lost interest in the surroundings and does not enjoy anything that she/he normally does. She/He does not want to go to college and tries to stay alone every time. She/He becomes sensitive and gets annoyed easily.

She/He finds it difficult to concentrate on her/his studies. Even the easiest topics are difficult for her/him to memorize. Her/His position and the marks are dropping day by day in the class. Ayesha/Ali finds it difficult to execute normal tasks and life for her/him became meaningless. She/He develops a negative approach towards everything as she/he always tries to criticize everything. She/He feels worthless and guilty.

Although Ayesha/Ali feels tired all the time, she/he has trouble sleeping at night. Her/His behaviour is unusual for her/his family. Her/His family has noticed that Ayesha/Ali is barely eating and has lost weight. Her/His family and friends are worried about her/him.

**Appendix B:**

**Table 1 Participants percentage who agreed (yes probably/yes definitely) with each causal attributes and care recommendations for mental illnesses (N=1328)**

| **1. Causal Attributes** | **N** | **%** |
| --- | --- | --- |
| **1. Socio-Economic causes** |  |  |
| Less educated parents | 639 | 48.1 |
| Father’s low wages | 750 | 56.5 |
| poverty | 898 | 67.6 |
| community disengagement | 684 | 51.5 |
| Negative attitudes tied to father's occupation | 677 | 51.0 |
| Family’s economic position | 867 | 65.3 |
| **2. Biomedical Causes** |  |  |
| Mental disorders | 828 | 62.3 |
| Brain disorders | 643 | 48.4 |
| Genetic issues | 530 | 39.9 |
| Drugs use | 405 | 30.5 |
| **3. Academic Stress** |  |  |
| Studies-related stress | 845 | 63.6 |
| Fear of Failure | 860 | 64.8 |
| Unmet family expectations in academics | 889 | 66.9 |
| Loss of community due to educational underachievement | 851 | 64.1 |
| **4. Psychosocial Causes** |  |  |
| Loneliness | 954 | 71.8 |
| Depression | 1005 | 75.7 |
| Major life events such as death of a loved one | 857 | 64.5 |
| Upbringing | 451 | 34.0 |
| Conflict with loved ones | 714 | 53.8 |
| Exposure to violence | 711 | 53.5 |
| Being bullied | 780 | 58.7 |
| **5. Religious and supernatural causes** |  |  |
| Guilt | 708 | 53.3 |
| Deviation from obligatory religious practices | 686 | 51.7 |
| God’s will | 838 | 63.1 |
| Perceived as divine retribution | 681 | 51.3 |
| Beliefs in sorcery, ghosts, and witchcraft | 458 | 34.5 |
| **6. Person-centered causes** |  |  |
| Developmental milestones in maturity | 895 | 67.4 |
| Sleeping, eating, and sports habits | 736 | 55.4 |
| **2. Care Recommendations** |  |  |
| **1. Informal Social Support** |  |  |
| Involving parents | 1001 | 75.4 |
| Family member | 855 | 64.4 |
| Friend | 997 | 75.1 |
| Peers with similar experiences | 968 | 72.9 |
| A confidential teacher or class teacher | 1019 | 76.7 |
| Fellow student | 1005 | 75.7 |
| **2. Formal Social Support from religious healers** |  |  |
| Religious healer | 665 | 50.1 |
| Faith healer | 751 | 56.6 |
| Traditional healer | 491 | 37.0 |
| Shamans | 331 | 24.9 |
| **3. Formal Support from health professionals** |  |  |
| Medication | 498 | 37.5 |
| Psychologist or psychiatrist | 807 | 60.8 |
| Homeopathy or acupuncture | 546 | 41.1 |
| Doctor (General practitioner) | 923 | 69.5 |
| **4. Self-Care** |  |  |
| Without doing anything | 333 | 25.1 |
| More self-discipline | 930 | 70.0 |
| Strict upbringing | 520 | 39.2 |
| Praying | 1084 | 81.7 |
| Leisure activities | 913 | 68.8 |
| Adjusting eating/sleeping habits | 1040 | 78.3 |

**Supplementary Material**

**Table 1: Mediation model: Indirect effect of causal attributes on the help-seeking care recommendations through the public perceived mental health stigma at Individual level variables**

|  | **Informal Social Support** | **Formal Social Support from Religious Healers** | **Formal support from health professionals** | **Self-Care** | |
| --- | --- | --- | --- | --- | --- |
| **A. Effect of Socio-economic causes** | | | | |  |
| **β(SE) *0.05, **0.01, ***0.001** | | | | |  |
| Socio-economic causes | 0.23(0.03)* | 0.21(0.03)*** | 0.19(0.03)*** | 0.2(0.02)*** | |
| Perceived Public Stereotypes | 0.03(0.02) | 0.05(0.02)** | 0.02(0.02) | 0.06(0.01)*** | |
| Age | 0.01(0.01) | -0.01(0.01) | -0.02(0.01) | 0.002(0.01) | |
| Female^a^ | 0.11(0.03)*** | -0.08(0.04)* | 0.14(0.04)*** | -0.01(0.03) | |
| Semi Urban^b^ | 0.16(0.04)*** | 0.05(0.05) | -0.01(0.05) | 0.05(0.03) | |
| Urban | 0.07(0.04) | 0.06(0.05) | -0.1(0.05)* | 0.1(0.04)** | |
| Joint^c^ | 0.05(0.04) | 0.08(0.04) | 0.07(0.04) | 0.05(0.03) | |
| Extended | 0.02(0.05) | 0.16(0.06)** | 0.03(0.06) | -0.04(0.04) | |
| Self-employed^d^ | -0.04(0.05) | -0.08(0.05) | -0.02(0.05) | -0.04(0.04) | |
| Government Job | 0.02(0.05) | -0.001(0.06) | 0.01(0.06) | -0.01(0.04) | |
| Private job | -0.02(0.07) | -0.07(0.07) | -0.09(0.07) | 0.03(0.05) | |
| Daily wage worker | -0.05(0.05) | -0.05(0.06) | -0.06(0.06) | -0.09(0.04)* | |
| **Indirect Effect** | **B {Boot SE (Boot 95%C.I)}** | | | | |
|  | 0.01{0.04(-0.001, 0.01)} | 0.01{0.03(0.001, 0.02)} | 0.003{0.004(-0.004, 0.01)} | 0.01{0.004(0.004, 0.02)} | |
| **B.** **Effect of Biomedical causes** | | | | |  |
| Biomedical Causes | 0.1(0.03)*** | 0.18(0.03)*** | 0.2(0.03)*** | 0.11(0.02) | |
| Perceived Public Stereotypes | 0.04(0.02)* | 0.06(0.02)** | 0.02(0.02) | 0.07(0.015) | |
| Age | 0.01(0.01) | -0.003(0.01) | -0.02(0.01) | 0.003(0.01) | |
| Female | 0.14(0.04)*** | -0.06(0.04) | 0.2(0.04)*** | 0.01(0.03) | |
| Semi Urban | 0.2(.04)*** | 0.06(0.05) | -0.01(0.1) | 0.05(0.04) | |
| Urban | 0.08(0.05) | 0.07(0.05) | -0.1(0.05)* | 0.11(0.04) | |
| Joint | 0.1(0.04) | 0.09(0.04)* | 0.08(0.04) | 0.06(0.03) | |
| Extended | 0.01(0.05) | 0.2(0.1)** | 0.03(0.06) | -0.04(0.04) | |
| Self-employed | -0.04(0.05) | -0.1(0.05) | -0.02(0.05) | -0.05(0.04) | |
| Government Job | 0.003(0.1) | -0.01(0.06) | 0.01(0.06) | -0.02(0.04) | |
| Private job | 0.02(0.1) | -0.03(0.07) | -0.05(0.07) | 0.06(0.05) | |
| Daily wage worker | -0.04(0.05) | -0.04(0.06) | -0.05(0.06) | -0.08(0.04) | |
| **Indirect Effect** | **B {Boot SE (Boot 95%C.I)}** | | | | |
|  | 0.01{0.003(0.001, 0.01)} | 0.01{0.004(0.001, 0.02)} | 0.003{0.003(-0.003, 0.01)} | 0.01{0.004(0.003, 0.02)} | |
| **C. Effect of Academic Stress** | | | | |  |
| Academic Stress | 0.21(0.02)*** | 0.12(0.03)*** | 0.13(0.03)*** | 0.15(0.02)*** | |
| Perceived Public Stereotypes | 0.03(0.02)* | 0.06(0.02)** | 0.02(0.02) | 0.06(0.01)*** | |
| Age | 0.01(0.01) | -0.01(0.01) | -0.02(0.01) | -0.0003(0.01) | |
| Female | 0.09(0.03)** | -0.08(0.04)* | 0.14(0.04)*** | -0.02(0.03) | |
| Semi Urban | 0.15(0.04)*** | 0.05(0.05) | -0.02(0.05) | 0.05(0.03) | |
| Urban | 0.08(0.04) | 0.06(0.05) | -0.1(0.05)* | 0.11(0.04)** | |
| Joint | 0.06(0.04) | 0.09(0.04)* | 0.08(0.04) | 0.05(0.03) | |
| Extended | 0.01(0.05) | 0.15(0.06)** | 0.02(0.06) | -0.05(0.04) | |
| Self-employed | -0.03(0.05) | -0.08(0.05) | -0.02(0.05) | -0.04(0.04) | |
| Government Job | 0.01(0.05) | -0.01(0.06) | 0.005(0.06) | -0.02(0.04) | |
| Private job | -0.01(0.06) | -0.06(0.07) | -0.08(0.07) | 0.04(0.05) | |
| Daily wage worker | -0.05(0.05) | -0.05(0.06) | -0.06(0.06) | -0.08(0.04)* | |
| **Indirect Effect** | **B {Boot SE (Boot 95%C.I)}** | | | | |
|  | 0.01{0.003(-0.0004, 0.01)} | 0.01{0.004(0.002, 0.02)} | 0.003{0.003(-0.003, 0.01)} | 0.009{0.003(0.003, 0.02)} | |
| **D. Effect of Psychosocial causes** | | | | |  |
| Psychosocial Causes | 0.29(0.0333)*** | 0.31(0.04)*** | 0.25(0.04)*** | 0.2(0.03)*** | |
| Perceived Public Stereotypes | 0.03(0.02) | 0.04(0.02)*** | 0.01(0.02) | 0.1(0.02)*** | |
| Age | 0.01(0.01) | -0.01(0.01)*** | -0.02(0.01) | 0.001(0.01) | |
| Female | 0.1(0.03)** | -0.1(0.04)* | 0.13(0.04)*** | -0.01(0.03) | |
| Semi Urban | 0.14(0.04)*** | 0.03(0.05)** | -0.03(0.05) | 0.03(0.04) | |
| Urban | 0.08(0.04) | 0.06(0.05)* | -0.1(0.05)* | 0.11(0.04)** | |
| Joint | 0.07(0.04) | 0.1(0.04)*** | 0.1(0.04) | 0.06(0.03) | |
| Extended | 0.03(0.05) | 0.2(0.06) | 0.04(0.06) | -0.04(0.04) | |
| Self-employed | -0.05(0.05) | -0.1(0.05) | -0.03(0.05) | -0.05(0.04) | |
| Government Job | -0.001(0.05) | -0.02(0.06) | -0.002(0.06) | -0.02(0.04) | |
| Private job | 0.001(0.07) | -0.05(0.0712) | -0.1(0.1) | 0.05(0.05) | |
| Daily wage worker | -0.05(0.05) | -0.06(0.06) | -0.06(0.06) | -0.09(0.04)* | |
| **Indirect Effect** | **B {Boot SE (Boot 95%C.I)}** | | | | |
|  | 0.01{0.01(-0.003, 0.02)} | 0.01{0.01(0.000, 0.03)} | 0.003{0.01(-0.01, 0.02)} | 0.02{0.01(0.01, 0.03)} | |
| **E. Effect of Religious and Supernatural causes** | | | | |  |
| Religious and Supernatural Causes | 0.21(0.03)*** | 0.3(0.03)*** | 0.2(0.03)*** | 0.23(0.02)*** | |
| Perceived Public Stereotypes | 0.03(0.02) | 0.04(0.02)* | 0.01(0.02) | 0.05(0.01)*** | |
| Age | 0.01(0.01)*** | -0.001(0.01) | -0.02(0.01) | 0.01(0.01) | |
| Female | 0.13(0.03)*** | -0.06(0.04) | 0.16(0.04)*** | 0.01(0.03) | |
| Semi Urban | 0.2(0.04) | 0.08(0.05) | 0.01(0.05) | 0.07(0.03)* | |
| Urban | 0.07(0.04) | 0.05(0.05) | -0.11(0.05)* | 0.1(0.04)** | |
| Joint | 0.05(0.04) | 0.07(0.04) | 0.07(0.04) | 0.04(0.03) | |
| Extended | -0.001(0.05) | 0.14(0.06)** | 0.01(0.06) | -0.06(0.04) | |
| Self-employed | -0.03(0.05) | -0.07(0.05) | -0.01(0.05) | -0.04(0.04) | |
| Government Job | 0.01(0.05) | -0.002(0.05) | 0.01(0.06) | -0.01(0.04) | |
| Private job | 0.02(0.07) | -0.03(0.07) | -0.06(0.07) | 0.06(0.05) | |
| Daily wage worker | -0.03(0.05) | -0.03(0.06) | -0.04(0.06) | -0.07(0.04) | |
| **Indirect Effect** | **B {Boot SE (Boot 95%C.I)}** | | | | |
|  | 0.01{0.004(-0.001, 0.01)} | 0.01{0.004(0.0004, 0.02)} | 0.002{0.004(-0.01, 0.01)} | 0.01{0.004(0.004, 0.02)} | |
| **F. Effect of Person Centred Causes** | | | | |  |
| Person Centred Causes | 0.11(0.02) | 0.1(0.02)*** | 0.1(0.02)*** | 0.14(0.02)*** | |
| Perceived Public Stereotypes | 0.05(0.02) | 0.1(0.02)*** | 0.03(0.02) | 0.1(0.01)*** | |
| Age | 0.01(0.01) | -0.005(0.01) | -0.02(0.01) | 0.001(0.01) | |
| Female^a^ | 0.2(0.03) | -0.05(0.04) | 0.2(0.04)*** | 0.02(0.03) | |
| Semi Urban^b^ | 0.2(0.04) | 0.06(0.05) | -0.01(0.05) | 0.05(0.03) | |
| Urban | 0.06(0.05) | 0.04(0.05) | -0.12(0.05)** | 0.09(0.04)* | |
| Joint^c^ | 0.06(0.04) | 0.08(0.04) | 0.07(0.04) | 0.05(0.03) | |
| Extended | 0.001(0.05) | 0.15(0.06)** | 0.02(0.06) | -0.06(0.04) | |
| Self-employed^d^ | -0.04(0.05) | -0.09(0.05) | -0.03(0.05) | -0.05(0.04) | |
| Government Job | -0.005(0.05) | -0.02(0.06) | -0.006(0.06) | -0.03(0.04) | |
| Private job | 0.0004(0.1) | -0.05(0.07) | -0.07(0.07) | 0.05(0.05) | |
| Daily wage worker | -0.05(0.05) | -0.05(0.06) | -0.06(0.06) | -0.08(0.04)* | |
| **Indirect Effect** | **B {Boot SE (Boot 95%C.I)}** | | | | |
|  | 0.0002{0.002(-0.003, 0.004)} | 0.0003{0.002(-0.01, 0.01)} | 0.0001{0.001(-0.002, 0.003)} | 0.0003{0.003(-0.005, 0.006)} | |

^a^reference Male

^b^reference Rural

^c^reference Nuclear

^d^reference Farmer
